# Reproductive and Gynecologic Effects of Glucagon-Like Peptide-1 Receptor Agonists in Women With Polycystic Ovary Syndrome: A Scoping Review

**DOI:** 10.7759/cureus.117072

**Published:** 2026-09-28

**Authors:** Jacklyn Snytte, Brielle Hazan, Amanda Weissman, Harvey N Mayrovitz

**Affiliations:** 1 Dr. Kiran C. Patel College of Osteopathic Medicine, Nova Southeastern University, Davie, USA; 2 Medical Education, Dr. Kiran C. Patel College of Allopathic Medicine, Nova Southeastern University, Davie, USA

**Keywords:** exenatide, fertility, glp-1 receptor agonists, hyperandrogenism, insulin resistance, liraglutide, menstrual irregularity, obesity, polycystic ovary syndrome, semaglutide

## Abstract

Polycystic ovary syndrome (PCOS) is a common endocrine disorder characterized by metabolic dysfunction, hyperandrogenism, ovulatory dysfunction, and infertility. Although glucagon-like peptide-1 receptor agonists (GLP-1 RAs) have demonstrated efficacy in promoting weight loss and improving insulin resistance, their effects on reproductive and gynecologic outcomes in women with PCOS have not been comprehensively summarized. This scoping review aimed to evaluate the available evidence regarding the reproductive and gynecologic outcomes associated with GLP-1 RA therapy in women with PCOS. A comprehensive literature search was conducted in PubMed, Embase, Web of Science, and Nursing & Allied Health Premium. Original studies evaluating GLP-1 RA therapy in women with PCOS and reporting reproductive or gynecologic outcomes were eligible for inclusion. Following duplicate removal, studies underwent title/abstract screening, full-text review, and standardized data extraction. Outcomes of interest included menstrual function, ovulation, fertility and pregnancy outcomes, hyperandrogenism, reproductive hormone profiles, ovarian function, and secondary metabolic outcomes. Twenty-eight studies met the inclusion criteria. Across the available literature, GLP-1 RA therapy was consistently associated with improvements in biochemical markers of hyperandrogenism, including reductions in total testosterone, free testosterone, free androgen index, and androstenedione, and increases in sex hormone-binding globulin. Improvements in menstrual regularity and reductions in amenorrhea were also frequently reported. Evidence regarding ovulation, fertility, and pregnancy outcomes was more limited but generally suggested improved ovulation rates and increased pregnancy rates in selected populations, particularly when GLP-1 RAs were combined with metformin. Nearly all included studies also demonstrated significant improvements in body weight, body mass index, and insulin resistance. Considerable heterogeneity in study design, interventions, and reported outcomes precluded quantitative synthesis. The overall findings suggest that GLP-1 RAs may provide reproductive and gynecologic benefits in women with PCOS in addition to their established metabolic effects. The most consistent improvements were observed in hyperandrogenism and menstrual function, whereas evidence supporting effects on fertility and pregnancy outcomes remains comparatively limited. Further high-quality prospective studies with standardized reproductive outcome measures are needed to better define the role of GLP-1 RAs in the management of PCOS.

## Introduction and background

Polycystic ovary syndrome (PCOS), recently renamed polyendocrine metabolic ovarian syndrome (PMOS), is one of the most common endocrine disorders affecting women of reproductive age [1]. PCOS affects up to 13% of reproductive-age women worldwide, often going undiagnosed [2,3]. The most common diagnostic criteria, named the Rotterdam criteria, characterize the disease by hyperandrogenism, ovulatory dysfunction, and polycystic ovarian morphology [4]. The disease is frequently associated with and closely linked to obesity, insulin resistance, dyslipidemia, and an increased risk of type 2 diabetes mellitus [5]. These metabolic abnormalities contribute to reproductive dysfunction as well as long-term cardiovascular risk. Currently, cornerstone treatment for overweight women with PCOS is lifestyle modification and weight loss, although sustained weight loss is often difficult to maintain. Due to this difficulty, pharmacological therapies to target metabolic dysfunction are being used more widely in PCOS management. Glucagon-like peptide-1 receptor agonists (GLP-1 RAs) have demonstrated substantial benefits in weight reduction and glycemic control and are increasingly prescribed for obesity and type 2 diabetes [6].

In addition to their metabolic benefits, GLP-1 RAs may affect reproductive and gynecologic outcomes in women with PCOS. Improvements in insulin resistance and weight loss may contribute to restoration of ovulation, menstrual regularity, reductions in hyperandrogenism, and improved fertility [7]. Improved insulin sensitivity may reduce ovarian androgen production and increase hepatic production of sex hormone-binding globulin (SHBG), thereby reducing circulating free androgens. Improvements in metabolic dysfunction may also contribute to restoration of ovulatory and menstrual function. These potential mechanisms provide a rationale for investigating whether GLP-1 RAs may influence reproductive outcomes in addition to their established metabolic effects.

Several reproductive and hormonal markers are commonly used to evaluate the reproductive manifestations of PCOS. Luteinizing hormone (LH) and follicle-stimulating hormone (FSH) reflect gonadotropin regulation and ovarian function, while SHBG influences the bioavailability of circulating androgens. Dehydroepiandrosterone sulfate (DHEAS) and androstenedione (A2) are circulating androgens that may contribute to the assessment of hyperandrogenism. The free androgen index (FAI), calculated using total testosterone and SHBG, provides an estimate of circulating free androgen activity. Changes in these hormonal parameters, along with clinical measures, such as hirsutism and menstrual cyclicity, can therefore be used to assess potential reproductive and gynecologic effects of GLP-1 RA therapy in women with PCOS.

The available literature is diverse, with studies differing in the interventions and reproductive endpoints measured. Several reviews have summarized the metabolic effects of GLP-1 RAs in PCOS [8,9], but to our knowledge, no scoping review has comprehensively mapped the evidence specifically addressing reproductive and gynecologic outcomes associated with these therapies. Therefore, the objective of this scoping review is to identify, characterize, and summarize the published literature evaluating reproductive and gynecologic outcomes following treatment with GLP-1 RAs in women with PCOS.

## Review

Methods

This review was performed using established methodological guidance for scoping reviews and reported in accordance with the Preferred Reporting Items for Systematic Reviews and Meta-Analyses extension for Scoping Reviews (PRISMA-ScR) [10].

Inclusion Criteria

Studies eligible for inclusion were those in which women were formally diagnosed with PCOS and were treated with GLP-1 RAs, including dual glucose-dependent insulinotropic polypeptide (GIP)/GLP-1 RAs. The study must also have reported at least one reproductive or gynecologic outcome, including but not limited to menstrual cyclicity, ovulation, fertility, pregnancy outcomes, hyperandrogenism, ovarian morphology, or reproductive hormone measurements. Studies evaluating GLP-1 RAs as monotherapy or in combination with other metabolic therapies, including sodium-glucose cotransporter-2 (SGLT2) inhibitors, were eligible for inclusion, provided that reproductive or gynecologic outcomes were reported.

Exclusion Criteria

Excluded were review articles, systematic reviews, meta-analyses, editorials, letters, conference abstracts without sufficient outcome data, study protocols, and studies without published results.

Protocol and Registration

No review protocol was registered or published for this scoping review.

Search Strategy

A comprehensive literature search was performed in PubMed, Embase, Web of Science, and Nursing & Allied Health Premium from 06/18/2026 through 06/22/2026. Searches were conducted using combinations of controlled vocabulary and free-text terms related to PCOS ("PCOS," "polycystic ovary syndrome") and GLP-1 RAs ("GLP-1," "glucagon-like peptide-1 receptor agonist," "liraglutide," "semaglutide," "exenatide"). The restrictions placed on study design during the initial search were English-language and peer-reviewed literature. The dates of the search were unlimited. The full search strategies for each database are provided in the Appendices.

Study Selection

Search results were exported from each database and imported into Rayyan software (Rayyan, Cambridge, MA) for citation management and duplicate removal. Following deduplication, two independent reviewers screened each title and abstract for eligibility. The first author participated in the review of all citations. Disagreements were resolved by a third reviewer, with unresolved conflicts adjudicated by the senior investigator. Studies meeting inclusion criteria based on title and abstract underwent full-text review using the same reviewer assignment process.

Data Extraction

Data from eligible studies were extracted using a standardized data extraction form. Extracted variables included study characteristics (author, publication year, country, and study design), participant characteristics, GLP-1 RA intervention, comparator, treatment duration, reproductive and gynecologic outcomes, and principal findings. Consistent with the scoping review methodology, a formal critical appraisal or risk-of-bias assessment of individual studies was not performed. The purpose of this review was to map the available evidence and characterize reported reproductive and gynecologic outcomes rather than to assess the certainty or quality of evidence.

Data Synthesis

Because of substantial heterogeneity in study design, intervention, comparator, treatment duration, and outcome definitions, findings were synthesized narratively. Extracted data were grouped according to reported reproductive and gynecologic outcomes, including menstrual function, fertility, pregnancy outcomes, hyperandrogenism, ovarian morphology, and reproductive hormone measurements. Metabolic outcomes were also summarized to provide clinical context. Given the heterogeneity of study designs, interventions, comparators, and outcome measures, quantitative effect estimates were not pooled. Individual study findings were summarized descriptively based on the reported direction and statistical significance of outcomes. The narrative synthesis was conducted in accordance with the principles outlined in the Synthesis Without Meta-analysis (SWiM) reporting guideline [11].

Results

Study Selection

A total of 1,416 records were identified through searches of PubMed, Embase, Web of Science, and Nursing & Allied Health Premium. After removal of 719 duplicate records, 697 unique articles underwent title and abstract screening. Following tier 1 screening, 76 full-text articles were assessed for eligibility. Of these 76, 28 met the inclusion criteria and are part of this review. The process is shown in Figure 1.

**Figure 1:**
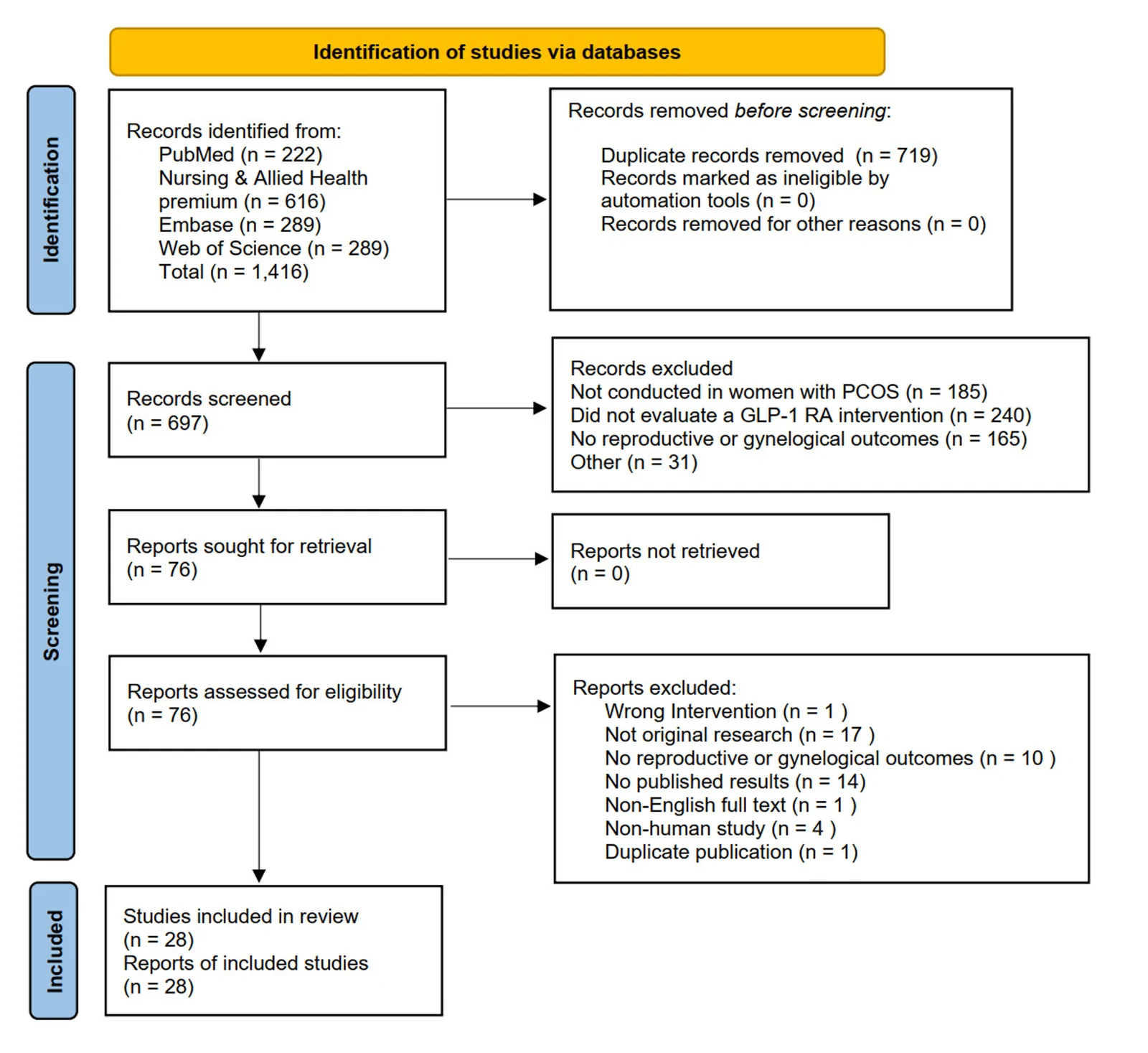
PRISMA 2020 flow diagram showing the study selection process. The figure is the PRISMA-ScR (Preferred Reporting Items for Systematic Reviews and Meta-Analyses extension for Scoping Reviews) 2020 flow diagram illustrating the identification, screening, eligibility assessment, and inclusion of studies evaluating the reproductive and gynecologic effects of glucagon-like peptide-1 receptor agonists (GLP-1 RAs) in women with polycystic ovary syndrome (PCOS).

Study Characteristics

The included studies evaluated a broad range of reproductive and gynecologic outcomes following treatment with GLP-1 RAs in women with PCOS. Frequently reported outcomes included menstrual cyclicity, ovulation, fertility and pregnancy outcomes, biochemical markers of hyperandrogenism, reproductive hormone profiles, and markers of ovarian function. Study designs included randomized controlled trials, prospective cohort studies, retrospective cohort studies, pilot studies, and case reports. Liraglutide (15 studies) was the most frequently investigated GLP-1 RA, followed by exenatide (five studies) and semaglutide (four studies). A summary of these studies and their key features is shown in Table 1.

**Table 1:** Study characteristics, interventions, and reproductive and gynecologic outcomes. The table summarizes the characteristics of studies included in this review, including study design, participant characteristics, interventions, comparators, treatment duration, and reported reproductive and gynecologic outcomes. A2, androstenedione; AFC, antral follicle count; AMH, anti-Müllerian hormone; DHEAS, dehydroepiandrosterone sulfate; E2, estradiol; FAI, free androgen index; FSH, follicle-stimulating hormone; GLP-1, glucagon-like peptide-1; GLP-1 RA, glucagon-like peptide-1 receptor agonist; HOMA-IR, homeostatic model assessment of insulin resistance; IVF, in vitro fertilization; LH, luteinizing hormone; MET, metformin; mFG, modified Ferriman-Gallwey score; NR, not reported; SGLT2, sodium-glucose cotransporter-2; SHBG, sex hormone-binding globulin; Free T, free testosterone; TT, total testosterone.

First author, year, country	Study design	Sample size	GLP-1 RA	Duration	Comparator	Reproductive/gynecologic outcomes	Main conclusions
Haiyan Chen, 2025, China [12]	Randomized controlled trial	100	Semaglutide	40 weeks	MET alone	Menstrual cycle recovery by 16 weeks, natural pregnancy weight by 40 weeks	Combination therapy alleviated menstrual irregularities and increased natural pregnancy rates
Fatimah Alawami, 2026, Ireland [13]	Retrospective cohort	58	Semaglutide	12 months	--	Androgens, gonadotropins, as well as reported changes in menstrual cycles, acne, and hirsutism	More regular menstrual cycles, improvement of acne and hirsutism
Maxim John Levy Barnett, 2025 [14]	Matched cohort study	9574	GLP-1 analogues	3 year	MET	Reproductive events (pregnancy, delivery, preeclampsia), endocrine changes (LH, FSH, androgen levels)	Decreased risk for amenorrhea and preeclampsia
Yuqin Zhang, 2023 [15]	Single-center randomized controlled trial	68	Dulaglutide	Time taken to achieve a 7% weight loss goal from baseline body weight, which was restricted to 6 months	Calorie-restricted diet alone	Changes in menstrual frequency, metabolic profiles, hormonal parameters	No significant change in menstrual frequency, other metabolic profiles, hormonal parameters, liver fat, and body composition
Chuan Xing, 2022, China [16]	Randomized controlled trial	60	Liraglutide	12 weeks	MET alone	Changes in menstrual frequency, TT, LH, FSH, progesterone, E2, and SHBG	Improvement in reproductive abnormalities and hyperandrogenemia; no significant changes in menstruation
Livadas S, 2020 [17]	Case report	5	Liraglutide	14 ± 4 months	--	Androgen levels, menstrual cycle pattern, hirsutism, and pregnancy	Significant decrease in insulin resistance index HOMA-IR and androgen levels, with significant improvement in fat deposition, menstrual cycle pattern, and the degree of hirsutism disclosed. One woman became pregnant during treatment
Craig C, 2014 [18]	Randomized pilot study	7	Exenatide	16 weeks	MET alone	Menstrual cyclicity	Improved ovulatory function as measured by increased menstrual cyclicity
Mojca Jensterle, 2015 [19]	Randomized controlled trial	45	Liraglutide	12 weeks	MET or roflumilast	LH, FSH, DHEAS, A2, TT, Free T, menstrual pattern	No significant differences were found in Free T, SHBG, A2, DHEAS, LH, FSH, or menstrual pattern
Mingyu Liao, 2024 [20]	Randomized controlled trial	60	Liraglutide	12 weeks	Cyproterone acetate/ethinylestradiol (CPA/EE) + MET	Menstrual cycle, polycystic ovaries, LH	Improved metabolic disorders and increased ovulation rates and LH
Karen E. Elkind-Hirsch, 2022 [21]	Randomized controlled trial	82	Liraglutide	32 weeks	Placebo	TT, DHEAS, menstrual frequency	Decreased FAI; menses occurrence was significantly increased
Mojca Jensterle Sever, 2014, Slovenia [22]	Randomized controlled trial	40	Liraglutide	12 weeks	MET alone and combination	A2, menstruation frequency	No significant changes in menstrual frequency or A2
Mainak Banerjee, 2026 [23]	Case reports	2	GLP-1 RA + SGLT2 inhibitor (Dulaglutide in case 1, Semaglutide in case 2)	Case 1: 7 months; Case 2: 5.5 months	--	Menstrual cycles, pregnancy	Menstrual normalization and accidental spontaneous conception
Malin Nylander, 2017, Denmark [24]	Randomized controlled trial	72	Liraglutide	26 weeks	Placebo	Bleeding ratio, ovarian volume, stromal volume, antral follicle count, Ferriman-Gallwey score, serum levels of AMH, SHBG, and androgens	Improvement in bleeding ratio and markers of ovarian function, ovarian volume decreased; increase in SHBG and decrease in Free T
Karen E Elkind-Hirsch, 2021 [25]	Randomized controlled trial	119	Exenatide (EQW)	24 weeks	Dapagliflozin (DAPA) alone and coadministered (EQW/DAPA), DAPA/extended-release (ER) metformin (DAPA/MET), and phentermine topiramate extended release (PHEN/TPM)	TT, FAI, conception (although not directly evaluated)	Decrease in TT levels and FAI were seen in all treatment groups. Four participants conceived during the trial, demonstrating return of ovulatory function
Daniel Fraser, 2025 [26]	Case report	1	Semaglutide, then tirzepatide	--	--	Pregnancy rate	Patient was found to be unexpectedly pregnant after the dramatic improvement of her symptoms
Vesna Salamun, 2018 [27]	Randomized controlled trial	28	Liraglutide	12 weeks	MET alone	Pregnancy rate	Increased pregnancy rates per embryo transfer and cumulative pregnancy rates
Andrej Janez, 2018 [28]	Randomized controlled trial	28	Liraglutide	12 weeks	MET alone	Pregnancy rate (IVF and IVF + spontaneous)	Increased pregnancy rate per embryo transfer and cumulative pregnancy rate
Qianying Yang, 2016 [29]	Case report	1	Exenatide	8 weeks	She had been treated with ovarian surgery, clomiphene citrate and gonadotropins, weight loss, and metformin, which have been effective for dominant follicle development; she still failed to conceive	Pregnancy	Improved ovarian function, promoted follicle development
Vesna Salamun, 2026 [30]	Cross-sectional study	20	Liraglutide	12 weeks	Placebo	Endometrial gene expression during the implantation window	Changes in the endometrial transcriptome that could potentially be important for improving endometrial receptivity and fertility
Mojca Jensterle, 2017, Slovenia [31]	Randomized controlled trial	30	Liraglutide	12 weeks	Liraglutide + MET	TT, A2, SHBG	Combination: Decrease in TT, Free T, A2; liraglutide: increased SHBG
Xue-Feng Long, 2025, China [32]	Randomized controlled trial	60	Liraglutide	12 weeks	MET and combination group	Free T, SHBG, LH, hyperandrogenemia	Combination group: improved the metabolic and reproductive endocrine characteristics
Qing Wen, 2023, China [33]	Randomized controlled trial	64	Beinaglutide	12 weeks	MET	Antral follicle count changes (via transvaginal three-dimensional ultrasound and transabdominal ultrasound) and TT, FSH, LH, LH/FSH ratio	TT and LH/FSH improved. No significant change in FSH, LH level, mFG score, ovarian volume, and AFC
Mojca Jensterle, 2015, Slovenia [34]	Pilot study	57	Liraglutide	12 weeks	--	LH, FSH, A2	No significant differences were found in LH, FSH, TT, Free T, SHBG, and A2
Tao Tao, 2020, China [35]	Randomized controlled trial	183	Exenatide	12 weeks	MET alone and combination	Hormonal changes (LH, FSH, Free T, FAI, SHBG, DHEAS, and A2)	Free T, SHBG, and A2 levels were all significantly decreased from baseline in each treatment group; no significant change in FSH and DHEAS
S. Frøssing, 2016 [36]	Randomized controlled trial	67	Liraglutide	26 weeks	Placebo	Free T, SHBG	Reduction in hyperandrogenism was demonstrated
Zhuoran Yang, 2026, China [37]	Randomized controlled trial	60	Tirzepatide	48 weeks (16 weeks of treatment)	MET alone	LH, FSH, Free T, SHBG, DHEAS, FAI, AMH, pregnancy rate	Improved reproductive endocrine parameters and menstrual cycle regularity, potential advantages in improving pregnancy outcomes
Xuefeng Long, 2023, China [38]	Retrospective study	102	Liraglutide	12 weeks	--	TT, Free T, FAI, LH	Significant decrease in TT, Free T, A2, FAI, LH, and LH/FSH ratio
Siyuan Zheng, 2017, China [39]	Randomized controlled trial	82	Exenatide	12 weeks	MET alone	Hirsutism (modified Ferriman-Gallwey scoring system), acne (Rosenfield score standard), SHBG, LH/FSH ratio	No significant difference in menstrual periods, modified Ferriman-Gallwey score, and Rosenfield score

Outcomes associated with GLP-1 RA use

Menstrual Outcomes

Menstrual function was among the most commonly evaluated reproductive outcomes, evaluated in 13 separate studies [12-24]. Three studies reported improvements in menstrual regularity or increased menstrual frequency following treatment with GLP-1 RAs, particularly among women who experienced significant weight loss (p < 0.01 [12,13]; p = 0.0001 [21]). One study also reported normalization in menstrual frequency following combination therapy with a GLP-1 RA and an SGLT2 inhibitor [23]. Restoration of regular menstrual cycles was observed in a substantial proportion of women receiving semaglutide, liraglutide, exenatide, and combination therapy with metformin, with one study showing an 86% improvement and 43% complete resumption of regularity [18]. Two studies demonstrated reductions in amenorrhea or improvements in bleeding patterns (p < 0.001 [14], p< 0.001 [24]), whereas others found no significant differences in menstrual outcomes between treatment groups [15,16,22]. Overall, the evidence suggests that GLP-1 RAs may improve menstrual function in women with PCOS, although findings were not uniform across all studies.

Fertility and Pregnancy Outcomes

Nine studies evaluated ovulatory function, fertility, or pregnancy-related outcomes [12,14,17,23,25-29]. Pregnancy rates included both spontaneous conception and conception after IVF treatment. Improvements in ovulation rates were reported following GLP-1 RA therapy, particularly when combined with metformin. Studies involving women undergoing assisted reproductive technologies demonstrated higher pregnancy rates following preconception treatment with liraglutide plus metformin compared with metformin alone (p < 0.05 [12], p = 0.03 [27]). One study observed spontaneous conception following combination GLP-1 RA and SGLT2 inhibitor therapy [23]. Additional reports described spontaneous conception following substantial weight loss associated with GLP-1 RA therapy, including individual case reports of unexpected pregnancies after treatment. However, fertility and pregnancy outcomes were assessed less frequently than other reproductive outcomes, and outcome measures varied considerably between studies.

Hyperandrogenism and Hormonal Outcomes

Hyperandrogenism was evaluated by 18 studies [13,15-17,19-22,24,25,30-37] and was the most consistently improved reproductive outcome. Multiple investigations reported significant reductions in total testosterone, free testosterone, FAI, A2, and LH following treatment with GLP-1 RAs. Several studies also demonstrated increases in SHBG and improvements in clinical manifestations of hyperandrogenism, including acne and hirsutism [13,17,24,37]. Improvements in Ferriman-Gallwey scores used to evaluate hirsutism were also reported. While reductions in androgen concentrations were commonly observed, changes in FSH, ovarian volume, and other endocrine parameters were less consistent. Some randomized trials found no significant differences in reproductive hormone profiles between treatment groups despite improvements in metabolic outcomes [33,34].

Ovarian Morphology Outcomes

Two studies evaluated markers of ovarian function and morphology, including antral follicle count changes, polycystic ovaries on ultrasound, and ovarian volume (p < 0.05 [20], p < 0.001 [24]). Improvements in ovarian function, follicular development, ovarian volume, and endometrial receptivity were reported following GLP-1 RA therapy. However, changes in antral follicle count and ovarian morphology were inconsistent, with several studies reporting no statistically significant differences after treatment.

Metabolic-Related Outcomes

Although reproductive and gynecologic outcomes were the primary focus of this review, improvements in metabolic parameters were reported in nearly all included studies. Significant reductions in body weight, body mass index, waist circumference, insulin resistance, and glucose metabolism were consistently observed. Several studies suggested that improvements in reproductive outcomes occurred alongside metabolic improvement, although the relationship between these changes was not directly evaluated.

Discussion

This scoping review synthesized the available evidence regarding the reproductive and gynecologic effects of GLP-1 RAs in women with PCOS. Overall, the findings suggest that GLP-1 RAs may provide reproductive benefits beyond their established metabolic effects. Across nearly all included studies, reproductive improvements occurred alongside significant reductions in body weight and improvements in insulin resistance.

Hyperandrogenism emerged as the reproductive outcome with the most consistent evidence of improvement. Multiple studies demonstrated significant reductions in total testosterone, free testosterone, FAI, A2, and LH following treatment with GLP-1 RAs. Several investigations also reported increases in SHBG and improvements in clinical manifestations of hyperandrogenism, including hirsutism and acne [13,17,24,37]. However, not all endocrine markers responded consistently, as changes in FSH, DHEAS, ovarian volume, and antral follicle count were less frequently observed or did not reach statistical significance [33,34]. These findings suggest that GLP-1 RAs may preferentially improve androgen excess rather than uniformly affecting all reproductive endocrine parameters.

Improvements in menstrual function were also commonly reported. Several studies demonstrated increased menstrual regularity, restoration of regular menstrual cycles, reductions in amenorrhea, or improved bleeding patterns following GLP-1 RA therapy [12,13,21,23]. Nevertheless, a small number of randomized trials reported no significant differences in menstrual outcomes between treatment groups despite improvements in metabolic measures and androgen profiles [15,16,22]. Differences in study populations, treatment duration, and methods used to assess menstrual function may contribute to these inconsistent findings.

Evidence regarding fertility and pregnancy outcomes was comparatively limited but encouraging. Several studies reported increased ovulation rates, spontaneous conception, or higher pregnancy rates following treatment with GLP-1 RAs, particularly when combined with metformin or used before assisted reproductive techniques [12,27]. Case reports also described spontaneous pregnancies following substantial weight loss during GLP-1 RA therapy [17,23,26]. However, fertility outcomes were infrequently designated as primary study endpoints, and several studies evaluated combination therapies, making it difficult to determine the independent contribution of GLP-1 RAs to these reproductive outcomes.

An important observation across the included literature was the close relationship between metabolic improvement and reproductive outcomes. Nearly every study reporting improvements in reproductive or gynecologic outcomes also demonstrated significant reductions in body weight, body mass index, insulin resistance, or other metabolic measures. While these findings support the role of GLP-1 RAs in improving the overall clinical profile of women with PCOS, they also make it difficult to determine whether improvements in reproductive function are direct pharmacologic effects or occur secondary to weight loss and improved metabolic health. Few studies were specifically designed to distinguish between these mechanisms.

Another notable finding was the substantial heterogeneity among the included studies. Investigations varied considerably in study design, sample size, treatment duration, comparator groups, GLP-1 RAs evaluated, and reproductive outcomes measured. Several studies examined GLP-1 RAs in combination with metformin or other glucose-lowering therapies, while others evaluated GLP-1 RA monotherapy. This variability limited direct comparison between studies and precluded quantitative synthesis of the available evidence.

Two recently published reviews also examined the reproductive effects of glucagon-like peptide-1 (GLP-1)-based therapies in women with PCOS and reached more cautious conclusions regarding the available evidence. Voros et al. and Forslund et al. highlighted the limited number of studies, heterogeneity of interventions and outcomes, and uncertainty regarding the independent reproductive effects of GLP-1-based therapies [40,41]. The present scoping review complements these analyses by mapping a broader range of reproductive and gynecologic outcomes across diverse study designs, while similarly demonstrating that evidence for fertility and pregnancy outcomes remains limited.

Strengths and limitations

This review has several strengths. A comprehensive search was performed across multiple electronic databases, and studies underwent duplicate removal, independent screening, and standardized data extraction. By focusing specifically on reproductive and gynecologic outcomes, this review provides a focused synthesis of an area in which the literature has historically emphasized metabolic endpoints. The inclusion of randomized controlled trials, observational studies, pilot studies, and case reports also provides a broad overview of the current evidence base.

Several limitations should be acknowledged. As a scoping review, this study was designed to map the existing literature rather than to evaluate treatment efficacy or conduct a quantitative meta-analysis. Many included studies enrolled relatively small numbers of participants, had short follow-up periods, or assessed reproductive outcomes as secondary endpoints. In addition, heterogeneity in outcome definitions and reporting limited direct comparison across studies. Several included studies evaluated GLP-1 RAs in combination with other metabolic therapies, including SGLT2 inhibitors and metformin, limiting the ability to isolate reproductive effects attributable specifically to GLP-1 receptor agonism. The review also included dual GIP/GLP-1 RAs alongside GLP-1 RAs because of their shared GLP-1 receptor activity and emerging use in metabolic management; however, findings from dual agonists should not be interpreted as evidence attributable exclusively to GLP-1 receptor agonism. The search was also restricted to English-language publications, which may have resulted in the omission of relevant studies published in other languages and may limit the generalizability of the findings. Finally, a formal critical appraisal or risk-of-bias assessment was not performed; therefore, the findings should be interpreted as a mapping of the available evidence rather than an assessment of the certainty or comparative quality of evidence.

Future research should prioritize adequately powered randomized controlled trials with standardized assessment of reproductive outcomes, including menstrual regularity, ovulation, fertility, pregnancy, and hormonal parameters. Studies designed to distinguish the independent reproductive effects of GLP-1 RAs from those mediated by weight loss would provide valuable insight into their role in the management of PCOS. Further investigation of newer GLP-1 RAs and dual incretin therapies, as well as long-term reproductive outcomes, is also warranted.

## Conclusions

GLP-1 RAs appear to offer reproductive and gynecologic benefits for women with PCOS in addition to their established metabolic effects. Across the available literature, the most consistent improvements were observed in biochemical markers of hyperandrogenism and menstrual function, while evidence supporting benefits in ovulation, fertility, and pregnancy outcomes remains comparatively limited. Although the current evidence is promising, considerable heterogeneity among studies and the frequent coexistence of substantial metabolic improvement make it difficult to determine the extent to which reproductive benefits are directly attributable to GLP-1 RA therapy. Future high-quality prospective studies with standardized reproductive outcome measures are needed to better define the role of these agents in the management of PCOS.

## Appendices

The full search strategies for each database

PubMed

("polycystic ovary syndrome" OR PCOS) AND ("GLP-1" OR "glucagon-like peptide-1 receptor agonist" OR liraglutide OR semaglutide OR exenatide)

Embase

("polycystic ovary syndrome" OR PCOS) AND ("GLP-1" OR "glucagon-like peptide-1 receptor agonist" OR liraglutide OR semaglutide OR exenatide)

Web of Science

("polycystic ovary syndrome" OR PCOS) AND ("GLP-1" OR "glucagon-like peptide-1 receptor agonist" OR liraglutide OR semaglutide OR exenatide)

Nursing & Allied Health Premium

("polycystic ovary syndrome" OR PCOS) AND ("GLP-1" OR "glucagon-like peptide-1 receptor agonist" OR liraglutide OR semaglutide OR exenatide)
